# Low-Carbon and Fundamental Properties of Eco-Efficient Mortar with Recycled Powders

**DOI:** 10.3390/ma14247503

**Published:** 2021-12-07

**Authors:** Chang Sun, Lulu Chen, Jianzhuang Xiao, Qiong Liu, Junqing Zuo

**Affiliations:** 1 School of Environment and Architecture, University of Shanghai for Science and Technology, Shanghai 200093, China; changsun@usst.edu.cn (C.S.); 193791864@st.usst.edu.cn (L.C.); lq612@usst.edu.cn (Q.L.); 2 National Engineering Technology Research Center for Prefabrication Construction in Civil Engineering, Tongji University, Shanghai 200092, China; 3 Department of Structural Engineering, College of Civil Engineering, Tongji University, Shanghai 200092, China; 4 Shanghai Construction Group Co., Ltd., Shanghai 200080, China; junqingzuo@163.com

**Keywords:** eco-efficient mortar, mechanical properties, long-term properties, carbon emission, analytic hierarchy process (AHP) method

## Abstract

Using recycled powders from solid waste is accepted as an effective strategy to realize the sustainable development of the construction industry. In our study, the cement was substituted by two kinds of recycled powders, i.e., spontaneous combustion gangue powder (SCGP) and recycled concrete powder (RCP), with a certain replacement ratio of 30%. The experimental variables were mainly the type of replacement powder (e.g., SCGP, RCP, and SCGP + RCP) and the grinding time of RCP (e.g., 25 min, 50 min, and 75 min). The fundamental properties, including mechanical properties, long-term properties, and carbon emission, were analyzed for all the mortar mixtures. Experimental results indicate that incorporation of RCP contributes to enhancing the toughness and dry shrinkage resistance of eco-efficient mortar, while SCGP positively affects the compressive strength and chloride resistance. The grinding process improves the activity of RCP to a certain extent, while a long grinding time leads to fusion and aggregation between powders. Investigation on CO_2_ emission demonstrates that carbon emission from cement production accounts for the largest proportion, 80~95%, in the total emission from mortar production. Combined with the AHP model, eco-efficient mortar containing 15% RCP ground for 50 min and 15% SCGP displays optimal fundamental properties.

## 1. Introduction

It is well known that irreversible CO_2_ emissions will negatively influence the economy, society, ecology, and many other aspects [1]. A series of strategies have been promoted by most countries, but the decarbonization process all over the world has achieved little [2,3]. CO_2_ emissions by the construction industry contribute greatly to the global CO_2_ emissions, as the production process of construction materials (e.g., cement and steel) consumes a lot of energy. For instance, it is estimated that by 2050, the annual global cement production will reach 8 billion tons [4] and the CO_2_ emissions from cement production will reach 6 billion tons [5]. Besides, with the development of urbanization, the defects of early urban planning and the functionality of buildings lead to the demolition of a large number of old buildings. The treatment of construction and demolition waste (CDW) is a hot issue at present [6]. The annual emission from CDW is over 2.3 billion tons in China [7], 0.7 billion tons in the United States [8], and 0.8 billion tons in the European Union [9]. At present, a large amount of CDW is still directly sent to landfills [10,11]. The proportion of CDW in landfills in China is not more than 50% [12], whereas that in the UK and Australia is about 44% and that in the United States is 29% [9,13,14]. Improper management of CDW has not only led to high CO_2_ emissions in the construction industry but also seriously polluted the environment [15,16,17]. Therefore, effective resource use of CDW is urgently needed. Meanwhile, similar straits exist in the treatment of industrial waste [18]. Abundant coal resources provide China and the European Union with sufficient energy supply [19], which also leads to huge coal gangue emissions. In China, the cumulative output of coal gangue has exceeded 500 million tons, which will increase by 350 million tons per year [20]. The accumulation of coal gangue not only has adverse effects on soil, the atmospheric environment, and the water environment but also leads to hazards, such as landslide, debris flow, and spontaneous combustion [21,22,23]. The main issue of the use of CDW and industrial solid waste is to make full use of the resource value of solid waste [24].

Recycling solid waste is a commonly accepted environmental-friendly way to depose of solid waste. There is a lot of mature research on the production of recycled coarse aggregate from crushed concrete waste or reusing solid waste in the subgrade cushion [25,26]. Previous studies have found that the old mortar attached to recycled concrete aggregate negatively affects the mechanical properties and durability of concrete containing recycled concrete aggregate [27,28,29]. The development of production technology and equipment of recycled concrete aggregate contribute to removing most of the old mortar. At the same time, the amount of recycled concrete powder (RCP) generated in the manufacturing process has been increased. It has been reported by Ma and Wang [30] that the amount of RCP accounts for 20–50% of the waste concrete in the production process. In RCP, the unhydrated cement particles still have hydration activity and the Ca(OH)_2_ generated by the initial hydration progress will react with SiO_2_ or Al_2_O_3_, which are beneficial to the pozzolanic reaction [31,32,33]. Therefore, RCP has great potential and value as a cementitious material. Research shows that unactivated RCP will lead to an obvious decrease in the mechanical properties of mortar [34,35]. Activated RCP can be a good substitute to replace part of cement, which has almost no negative impact [31,36]. However, the activation process is always accompanied by energy consumption and the maximum replacement of cement will be limited.

Some studies have found that the incorporation of pozzolanic material and inert particles at an appropriate ratio can improve the activity of the binder material [37,38,39]. Inert particles contribute to the dispersion of cementitious materials, which provides sufficient space for the chemical reaction and nucleation sites for the formation of hydration products. The high content of clay minerals in coal gangue leads to a good pozzolanic activity of the powder from coal gangue [40]. The feasibility of using coal gangue powder as supplementary cementitious materials (SCMs) has been confirmed [41,42]. The authors [35] found that the compounds found in spontaneous combustion gangue powder (SCGP) and RCP lead to acceptable mechanical properties of mortar. Although there are some research results on the use of CDW and coal gangue waste, the mixture proportion with relatively superior material properties and environmental impact still needs to be studied.

In this study, different mixing methods of RCP and SCGP were studied to explore the affecting mechanism of RCP and SCGP on mortar properties. The effect of different grinding times on the performance of eco-efficient mortar was studied to investigate the appropriate manufacturing process of RCP. Combined with the analytical hierarchy process (AHP) method, the fundamental performance of eco-efficient mortar was evaluated and analyzed, which includes mechanical properties (e.g., compressive strength and flexural strength), long-term properties (e.g., chloride resistance and dry shrinkage), and environmental benefits (carbon emissions).

## 2. Materials and Methods

### 2.1. Materials

Portland cement (PC) labeled P.C. 42.5 in accordance with GB175-2007 [43] was used in this experiment. River sand with a density of 2.7 g/cm^3^ was used as fine aggregate. The chemical composition of sand follows Chinese code GB/T17671-1999 [44], where the content of SiO_2_ is not less than 98%. The chemical compositions and physical properties of cementitious materials (e.g., PC, SCGP, and RCP) are listed in Table 1. SCGP and RCP were used as binder materials to substitute part of the cement. SCGP was manufactured by a factory in Shanxi Province. SCGP was ground from spontaneous combustion coal gangue. Coal gangue is a kind of solid waste produced during coal mining, which contains an amount of carbon. The spontaneous combustion coal gangue used in this study had been stacked for a long time, and the content of carbon was reduced after spontaneous combustion.

**Figure 1:**
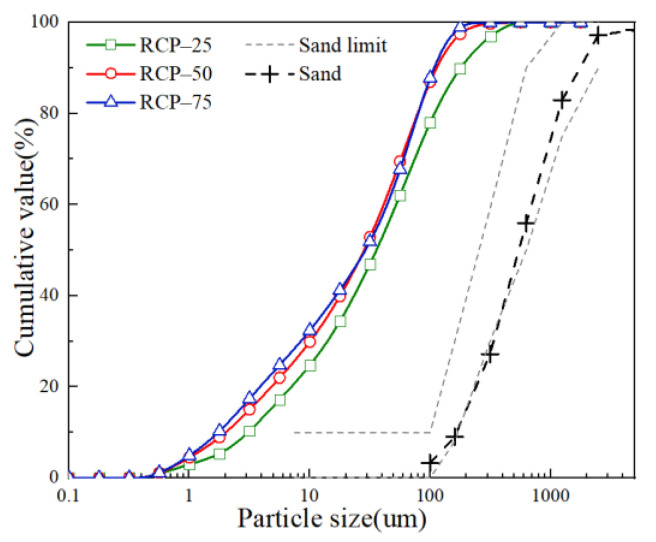
Particle size distribution for RCP and fine aggregate.

RCP was produced in the laboratory. The source material for RCP was provided by Shanghai Youhong Environmental Protection Technology Co. Ltd., Shanghai, China. Two kinds of recycled aggregate (recycled coarse aggregate and recycled fine aggregate) are produced from the demolished waste concrete after a series of processes in the factory. The recycled fine aggregates (RFAs) with particle size under 5 mm were used to manufacture RCP in this study. Before the grinding process, RFAs with particle sizes less than 0.3 mm were removed by sieving to avoid the influence of dust on RCP. The mill in this test was a kind of ball mill for cement. The storage barrel was a cylinder of ϕ 500 × 500 mm, the grinding media were 100 kg steel balls of different shapes. The rotation speed of the mill was 48 r/min, and 5 kg of raw material for RCP was added each time. The milling time of RCP varied in this study, such as 25 min, 50 min, and 75 min, to explore suitable RCP processing. The particle size distribution curves for the RCP under different grinding times are shown in Figure 1. According to Figure 1, the proportions of RCP particles under 100 um for RCP with different grinding times behave in the order: RCP_50min_ ≈ RCP_75min_ > RCP_25min_.

### 2.2. Mortar Mixture

Experimental results have pointed out that the replacement ratio of cement by SCMs should be restricted to a certain value to maintain acceptable properties of mortar or concrete, such as 30% [35]. In this study, the substitution ratio for cement was kept at 30% for all the mixtures to analyze the optimum mixing ratio of RCP and SCGP. Table 2 lists two factors considered in the mixture proportions design: (a) SCM composition, e.g., RCP, SCGP, and RCP + SCGP (RCP:SCGP = 1:1 by mass) and (b) RCP grinding time, e.g., 25 min, 50 min, and 75 min.

Total five mortar mixes were designed in accordance with the Chinese standard JGJ55-2011 [45], as shown in Table 3. RM1-RM3 were mixtures considering factor A, while RM3-RM5 considered factor B. The mix ID represents the substitution ratio of SCMs and the milling time for RCP. For example, the mix ID M-15R_25_-15S expresses the mortar mixture with 15% RCP (under 25 min milling) and 15% SCGP. The water-to-binder ratio and the binder-to-sand ratio for all five mixtures were 0.40 and 0.54, respectively. The contents of RCP and SCGP were calculated by the following formula:

(1) $$\frac{M_{cement}}{M_{RCP}}=\frac{1-\varphi_{RCP}-\varphi_{SCGP}}{\varphi_{RCP}}$$

(2) $$\frac{M_{cement}}{M_{SCGP}}=\frac{1-\varphi_{RCP}-\varphi_{SCGP}}{\varphi_{SCGP}}$$

where, $M_{cement}$, $M_{RCP}$, and $M_{SCGP}$ represent the contents of cement, RCP, and SCGP; $\varphi_{RCP}{\;\mathrm{and}\;\varphi }_{SCGP}$ are the replacement ratios of RCP and SCGP, respectively.

The preparation and curing of specimens are conducted in compliance with the Chinese standard JGJ-T70-2009 [46].

### 2.3. Test Methods

#### 2.3.1. Mechanical Properties Tests

The mechanical properties of eco-efficient mortar were evaluated by compressive strength and flexural strength. Referring to the Chinese standard GB/T17671-1999 [44], compressive and flexural strength tests were conducted on specimens of varied curing ages. The flexural strength was measured by three prisms with dimensions of 40 mm × 40 mm × 160 mm. After the flexural strength test, six pieces of broken prisms were used to test the compressive strength.

#### 2.3.2. Long-Term Properties Tests

**Figure 2:**
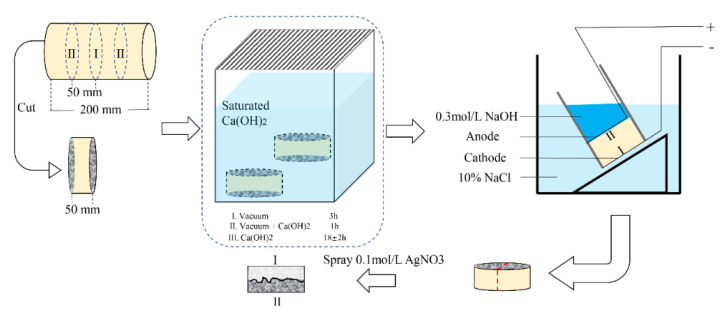
RCM test procedures.

The chloride migration properties and dry shrinkage of mortar were tested to evaluate the long-term properties of mortar. The rapid chloride migration (RCM) test was conducted in compliance with the Chinese standard GB/T50082-2009 [47]. The RCM test procedure is illustrated in Figure 2. The non-steady-state diffusion coefficient of chloride (*D_RCM_*) was calculated from the following equation:

(3) $$D_{RCM}=\frac{0.0239\times \left(273+T\right)L}{\left(U-2\right)t}\left(X_{d}-0.0238\sqrt{\frac{273+TLX_{d}}{U-2}}\right)$$

where *X_d_* is the average chloride ion penetration depth (mm), *U* is the applied voltage (v), *T* is the average value of the initial and final temperatures (°C) in the anolyte solution, *L* is the thickness of the sample (mm), and *t* is the test duration (h).

According to ASTM C157/C157M-08 [48] and JGJ-T70-2009 [46], the 25 mm × 25 mm × 280 mm prisms were used to measure the dry shrinkage of mortar. Firstly, the prisms were cured in the molds for 7 days, at the temperature of 20 ± 2 ℃ and the relative humidity of over 90%. Secondly, the specimens were demolded and the initial length (L_i_) was measured for each specimen. Then, the specimens were moved to the dry shrinkage curing box, with a temperature of 20 ± 2 °C and a relative humidity of 60 ± 5%. The specimens were measured at different ages until cured in the dry shrinkage curing box for 28 days.

(4) $$L\left(\mu \varepsilon \right)=\frac{L_{x}-L_{i}}{L_{g}}$$

where *L* is the change in length (*με*), *L_x_* is the length of the specimen (mm) at age *x*, *L_i_* is the initial length of the specimen (mm), and *L_g_* is the gauge length (mm).

## 3. Results and Discussions

### 3.1. Performance of Eco-Efficient Mortar

#### 3.1.1. Performance of Eco-Efficient Mortar in Series A

**Figure 3:**
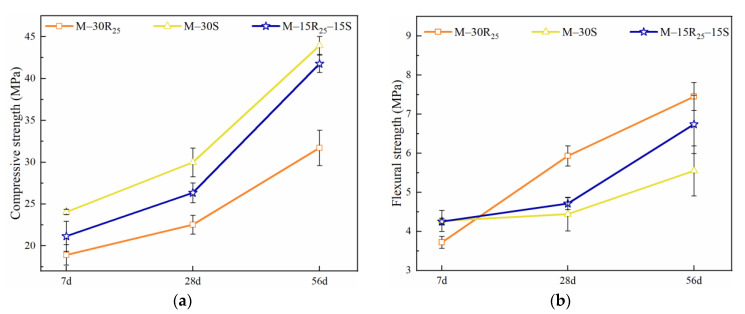
Mechanical strength of eco-efficient mortar mixtures in Series A: (**a**) compressive strength, (**b**) flexural strength.

The compressive strength is the main index to measure the mechanical properties of mortar. Figure 3 shows that the compressive strength of eco-efficient mortar in Series A is in the order M-30S > M-15R_25_-15S > M-30R_25_. The test results reveal that the pozzolanic activity of SCGP is superior to that of RCP. When the curing age increases from 7 days to 28 days, the increase in compressive strength for M-30R_25_, M-30S, and M-15R_25_-15S is 19.08%, 24.66%, and 24.54%, respectively. The increase for M-30R_25_ is the lowest in Series A, which results from the low content of hydration products generated as 30% of cement was substituted by RCP with relatively lower pozzolanic activity. According to ASTM C618 [49], the pozzolanic activity of SCMs is related to the content of Si and Al. As listed in Table 1, the content of Si and Al in RCP is lower than that in SCGP, which results in the relatively lower pozzolanic activity of RCP. As the curing period increases from 28 to 56 days, the compressive strength growth of M-30R_25_, M-30S, and M-15R_25_-15S are 40.79%, 46.60%, and 58.58%, respectively. After 28 days of curing, the hydration process of cement has been basically completed and pozzolanic reaction contributes a lot to the growth in compressive strength. Among Series A, the increase for M-15R_25_-15S is higher than that for M-30S, which benefits from the synergetic effect of RCP and SCGP. From Table 1, it can be found that the specific surface area of RCP is larger than that of SCGP and PC, which leads to the higher water content of mortar using RCP compared to mixtures without RCP after leaving the standard curing condition. The water inside the mortar will promote the second hydration action and lead to a more compact microstructure and an obvious increase in the compressive strength after 28 days of curing. A similar conclusion has been drawn by Menéndez et al. [37] and Ghrici et al. [50] that the ternary cementitious material system is beneficial for promoting secondary hydration in mortar and concrete.

The flexural strengths of the mortar mixtures in Series A are illustrated in Figure 3b. Comparing Figure 3a,b, it can be observed that the effect of SCMs on flexural strength is different from their effect on compressive strength. The flexural strength of M-30R_25_ at 7 days is lower than that of M-30S and M-15R_25_-15S, while after 28 and 56 days of curing, the flexural strength of M-30R_25_ is the highest among Series A. The experimental results reveal that in contrast to the effect on the compressive strength, using RCP will positively affect the flexural strength and toughness of eco-efficient mortar. The positive effect of RCP on the flexural strength is highlighted after 28 days of curing, which is attributed to the prolonged cement hydration process caused by the micro-aggregate effect. Mao et al. [51] have confirmed that an improvement in the toughness of concrete can be seen when the recycled powder replacement ratio is less than 30%. Consequently, the incorporation of SCGP contributes to increasing the compressive strength and the use of RCP is effective in improving the flexural strength.

**Figure 4:**
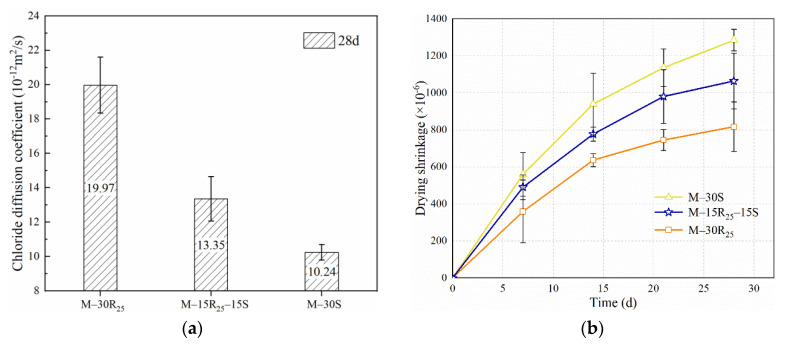
Durability results of eco-efficient mortar in Series A: (**a**) chloride diffusion coefficient (*D_RCM_*) and (**b**) drying shrinkage.

After a curing period of 28 days, the effects of SCMs (e.g., RCP, SCGP, and RCP + SCGP) on the chloride diffusion coefficient (*D_RCM_*) are presented in Figure 4. Experimental results indicate that the value of *D_RCM_* for M-30S is the lowest in Series A. A similar conclusion has been drawn by Elfmarkova et al. [52], that the incorporation of pozzolanic material leads to an improvement in the chloride resistance ability of mortar. The chloride diffusion coefficient is related to pores with a diameter in the range of 10–10^4^ nm [53,54]. The positive effect on the pores (10~10^4^ nm) by SCGP is attributed to the compact structure resulting from the second-hydration action. In addition, the chloride diffusion coefficient is influenced by hydration products with different chloride binding mechanisms [55]. Researchers have found that SCMs containing aluminum result in the formation of Friedel’s salt (C_3_A·CaC_l2_·10H_2_O), which is considered to absorb chloride ions [56,57]. According to Table 1, the Al_2_O_3_ content in SCGP is higher than in PC and RCP, which will contribute to enhancing the chloride binding ability. Consequently, resulting from the positive effect of SCGP on the microstructure and the chloride ion binding capacity, the eco-efficient mortar using SCGP shows good chloride resistance ability.

Figure 4b shows the dry shrinkage of eco-efficient mortar in Series A. The dry shrinkage strain at 28 days for specimens in Series A ranges from 800 to 1300 με. The value of dry shrinkage strain has been commonly considered to relate to compressive strength. From Figure 4b, in Series A, the dry shrinkage strain of M-30R_25_ has been observed to be the lowest across the curing age, which is consistent with the results of 7-day and 28-day compressive strength. The low dry shrinkage strain of M-30R_25_ mainly results from the adverse effect of RCP, with relatively low activity on the hydration process of binder materials. In M-30R_25_, incorporating RCP to replace 30% of cement leads to a decrease in the amounts of hydration products, including C–S–H. Benefiting from incomplete hydration, more macropores with a large volume-to-surface ratio are generated in M-30R_25_, which leads to relatively small capillary stress and small shrinkage. Furthermore, the unactive particles in RCP with filling ability contribute a lot to resisting the capillary stress caused by water loss and inhibit shrinkage [58,59,60]. In addition, it is observed from Figure 4b that the shrinkage of M-30S is higher compared to that of M-30R_25_. This phenomenon results from the second hydration caused by the incorporation of SCGP with relatively high pozzolanic activity [35]. The second hydration will lead to a lower proportion of macropores and increased microporosities of C–S–H. Under the same humidity conditions, the size of the pore diameter is the main reason for moisture loss [61]. The increase in the number of small capillary pores results in greater capillary pressure and a larger shrinkage. In conclusion, using SCGP leads to an increase in shrinkage and incorporating RCP will inhibit shrinkage. It can be found from Figure 4b that the shrinkage strain of the M-15R_25_-15S is between the values of M-30R_25_ and M-30S, indicating that a synergistic effect between SCGP and RCP still positively affects the dry shrinkage. Before 7 days of curing, the early dry shrinkage of M-15R_25_-15S is similar to that of M-30S. After 7 days, the inhibition of RCP gradually slows down the increase in shrinkage for M-15R_25_-15S, which is attributed to the physical resistance caused by RCP particles.

#### 3.1.2. Performance of Eco-Efficient Mortar in Series B

**Figure 5:**
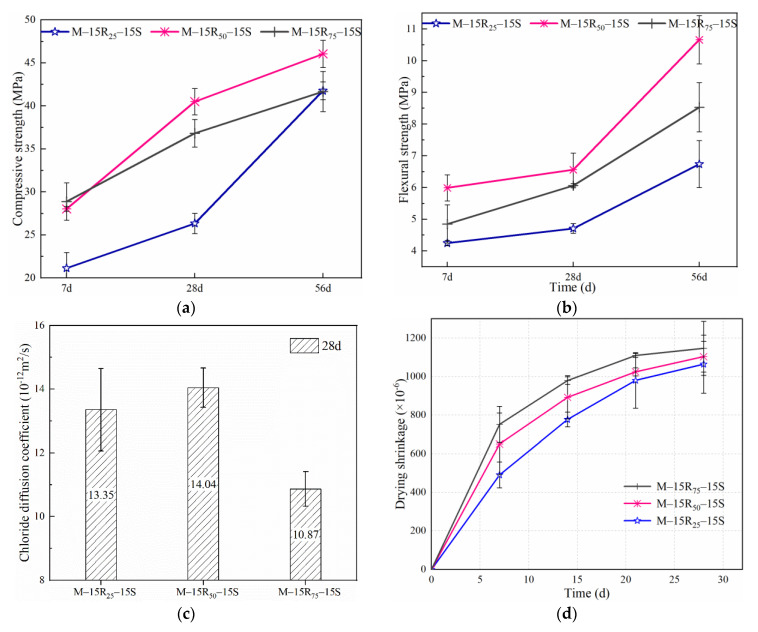
Effect of RCP grinding time on properties of eco-efficient mortar in Series B: (**a**) compressive strength, (**b**) flexural strength, (**c**) chloride migration coefficient, and (**d**) drying shrinkage.

Figure 5 shows the effect of RCP with different grinding times on various properties of eco-efficient mortar in Series B. When grinding time increases from 25 to 50 min, an obvious increase in the mechanical properties at 7–56 days can be observed from Figure 5a,b. When the grinding time exceeds 50 min, the positive effect of the finely grinding process on the properties of RCP and eco-efficient mortar is weakened. As shown in Figure 5a,b, the compressive and flexural strengths of M-15R_75_-15S are lower than those of M-15R_50_-15S. It is difficult to finely grind RCP after a certain grinding time, as Figure 1 shows that the particle size distribution of RCP under grinding of 50 and 75 min is similar. Besides, long-time grinding will lead to aggregation and fusion between particles and mix the impurities from equipment wear [62,63]. Grinding RCP for 50 min is appropriate only considering the mechanical properties of mortar.

With an increase in grinding time, there is no obvious regularity in long-term performance. As per Figure 5c, with an increase in the grinding time from 25 to 50 min, the value of *D_RCM_* increases by 5.17%; while a reduction of 18.58% in *D_RCM_* is observed on increasing the grinding time from 25 to 75 min. As shown in Figure 1, the proportion of RCP particles under 100 um for RCP_50min_ is relatively high. Incorporating RCP (under 50 min of grinding) with a smaller particle size introduces weak points in eco-efficient mortar under chloride attack, which results from the high water absorption ratio and the gap due to particle stacking. Therefore, the chloride resistance of M-15R_50_-15S decreases slightly relative to that of M-15R_25_-15S. As mentioned in research by Yang. et al. [64] and Kumar. et al. [65], after 75 min of grinding, the aggregation and fusion among RCP will modify the surface smoothness of RCP particles and reduce the porosity. The RCP particle with a smoother surface and lower porosity will contribute to the better distribution of cement and further enhance water resistance and chloride resistance [66]. It can be seen from Figure 5d that the drying shrinkage of the eco-efficient mortar increases with the grinding time of RCP. The difference in shrinkage strain among Series B mainly appeared at an early age. Finer particles of RCP under 50 min of grinding can aggravate cement hydration, beneficial to the microaggregate effect, which leads to an increase in the shrinkage strain. In addition, RCP particles after 75 min of grinding can further enhance the shrinkage strain due to the positive effect on cement dispersion after aggregation and fusion. Therefore, the shrinkage value is noticed in the order M-15R_75_-15S > M-15R_50_-15S > M-15R_25_-15S. As the physical grinding has no obvious excitation effect on the pozzolanic activity of RCP, the values of the 28-day shrinkage strain in Series B are similar.

### 3.2. Fundamental Analysis

In addition to the effect on the performance of eco-efficient mortar, the incorporation of recycled material has positive environmental impacts. Considering the environmental effects (CO_2_ emission) and performance (e.g., compressive strength, flexural strength, chloride resistance, and dry shrinkage) of different mixtures, the optimum mixture proportion will be investigated in this study. The CO_2_ emission in the study represents only carbon dioxide rather than a carbon dioxide equivalent.

As everyone knows, excessive emission of greenhouse gases is the main factor causing global warming and CO_2_ emissions account for 82% of the total greenhouse gas emissions [67]. China’s 14th five-year plan for the development of a circular economy [12] and the European Commission’s Circular Economy Package [68] clearly announced the plan to reduce CO_2_ emissions. Therefore, for construction materials with huge annual output, such as mortar and concrete, their CO_2_ emissions also deserve attention.

#### 3.2.1. CO_2_ Emissions Analysis

RCP is manufactured in the laboratory, and the CO_2_ emission from RCP mainly depends on the energy consumption in the grinding process. Liu et al. [69] have pointed out that the electric energy consumed by grinding RCP for 25, 50, and 75 min is of the order 0.27, 0.52, and 0.77 kW·h/kg, respectively. Yu et al. [70] have stated that 1 kW·h electric energy consumption will release 875 g of CO_2_. In this study, p*_i_* represents the CO_2_ emissions from the production process of raw materials. Accordingly, the values of p*_RCP_* of 25, 50, and 75 min are 236.25, 455.00, and 673.75 kg/t, respectively.

**Figure 6:**
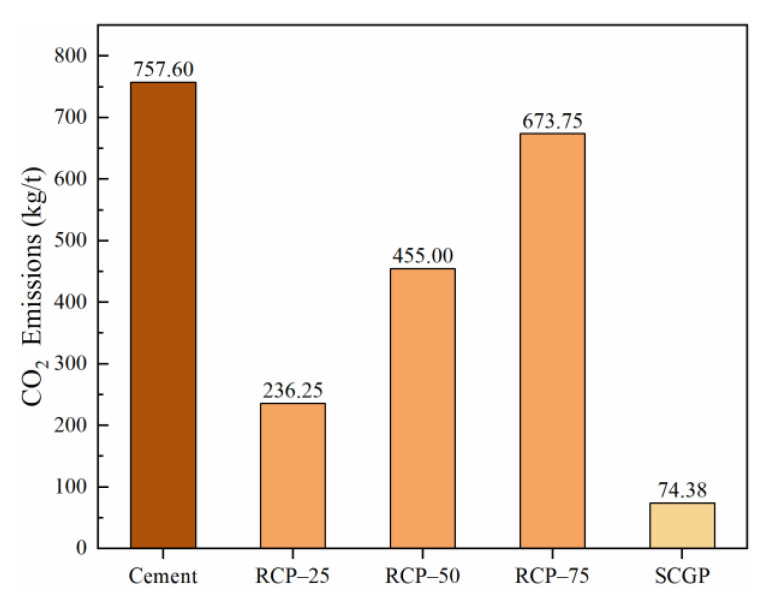
CO_2_ emissions from the production process (p_i_) of a binder material.

SCGP is a product of the factory, and its production energy consumption is determined as 0.085 kW·h/kg according to the power of the professional grinder. As proposed by Yu et al. [70], the value of p*_SCGP_* is 74.38 kg/t. Xiao et al. [5] have pointed out that p*_Sand_* is 0.649 kg/t and p*_Cement_* is 757.60 kg/t. The value of p*_Water_* is considered as zero in this model, as the CO_2_ emission from water is negligible [71,72]. CO_2_ emissions from the production of different binder materials (p*_i_*) are shown in Figure 6. The CO_2_ emission from cement production is extremely high, almost 3.21 times as the CO_2_ emission from grinding RCP (p*_RCP-25_*) and 10.19 times as the value of p*_SCGP_* with the mature production process. Researchers have confirmed that the grinding process contributes to increasing the surface area of RCP and exposes the non-carbonized part in the building demolition. The increase in the CO_2_ absorption of eco-efficient mortar can largely offset the CO_2_ emissions from grinding [5,73]. Therefore, stimulating the potential of solid waste to meet the basic requirements of general cementitious materials is an effective method to reduce carbon emissions by the cement industry.

In addition to raw material production, material transportation will also consume part of CO_2_ [74]. The CO_2_ emission generated by transportation (t_i_) is related to the transportation distance of each material and the CO_2_ generated by the corresponding transportation mode. The data of transportation distance and CO_2_ emission by different transportation modes are derived from Xiao et al. [5], listed in Table 4. Referring to the investigation by Xiao et al. [5], for each ton of solid waste reused instead of consigned to a landfill, the CO_2_ emission will be reduced by 1.055 kg. The use of RCP or SCGP contributes to reducing the amount of solid waste ending up in a landfill, as a result, reducing CO_2_ emission by 1.055 kg/t. Similar to RCP, the transportation process of SCGP also includes two stages: (1) massive coal gangue is transported to the factory and (2) the SCGP product is transported out for sale. Among them, the transportation of RCP and SCGP emits more CO_2_, but the transportation of solid waste to landfills also emits CO_2_. The average transport distance of solid waste to a landfill is 30 km. Medium-heavy trucks are used for transportation, which produce 4.523 kg/t CO_2_. CO_2_ emission from the transportation to landfills should be considered when measuring the actual CO_2_ emissions from RCP and SCGP on transportation.

To quantify the environmental impact of different recycled mortars, refer to the research by McLellan et al. [75]. Equation (5), on the amount of CO_2_ ($f_{CO_{2}}$) produced by per m^3^ of recycled mortar, is proposed as following:

(5) $$f_{CO_{2}}=\overset{n}{\underset{i=1}{\sum }}m_{i}\left(p_{i}+t_{i}-LD_{i}-TD_{i}\right)$$

where $f_{CO_{2}}$ is the total CO_2_ emissions (kg/t), m*_i_* is the mass of component *i*, p*_i_* = CO_2_ emissions per unit ton of *i* manufactured (kg/t), $t_{i}$ = CO_2_ emissions per unit ton of *i* transported (kg/t), $LD_{i}\;$= CO_2_ emissions amount per ton of solid waste landfill (kg/t), and $TD_{i}$ = CO_2_ emissions from the transportation of solid waste (kg/t).

**Figure 7:**
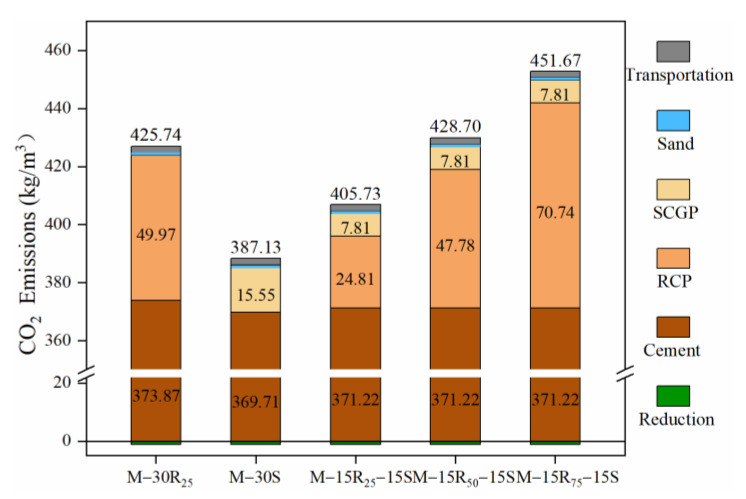
CO_2_ emission from all eco-efficient mortar mixtures.

The total CO_2_ emission by all eco-efficient mortar mixtures is shown in Figure 7. Figure 7 shows that the production of raw materials is the main cause of CO_2_ emission and the CO_2_ emission from cement production plays a major role in it. CO_2_ emission from cement production accounts for 80~95% of the total emission from mortar production for RM1~RM5. It is also found that the difference in CO_2_ emission from eco-efficient mortar in Series B is due to the grinding time of RCP. Using solid waste to substitute part of cement will achieve not only disposal of solid waste resources but also reduction in CO_2_ emission. As the production technology will be optimized gradually in the future, the manufacturing process of solid waste will achieve less CO_2_ emission. Figure 7 includes the reduction in CO_2_ emissions from solid waste landfills and solid waste transportation to landfills.

#### 3.2.2. AHP Model

**Figure 8:**
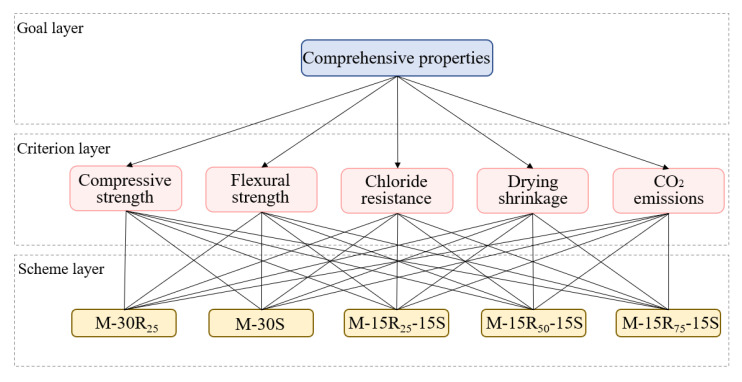
AHP model for fundamental properties of mortar.

The AHP [76] is an effective qualitative multi-objective fundamental evaluation method. This study investigates the eco-efficient mortar mixture proportion with the best fundamental performance and environmental benefits using the AHP method. As shown in Figure 8, the goal layer of this model is the fundamental properties of mortar. The criterion layer includes five factors: compressive strength, flexural strength, chloride resistance, dry shrinkage resistance, and CO_2_ emission. The scheme layer contains all the eco-efficient mortar mixtures in this study. According to the judgment criteria proposed by Saaty [76], the fundamental scale for the importance of all factors in the criterion layer is given in Table 5. In combination with experimental results, the fundamental properties of all the mixtures will be evaluated.

Judging from previous experience, compressive strength is regarded as the most important factor; the importance of dry shrinkage and carbon emission is second only to that of compressive strength. The importance of chloride resistance is moderate, and the importance of flexural strength is the least. The matrix of five factors that is based on the fundamental scale by Saaty [76] is listed in Table 6. The matrix has been confirmed to meet the requirements, that is, the value of the consistency ratio (CR) is 0.0115, smaller than 0.1. Then, the matrix is normalized and the scale of priorities (or weights) is obtained by the arithmetic average method, as shown in Table 6.

#### 3.2.3. Optimized Mixtures Proportion Analysis

To quantitatively analyze the fundamental performance of each mixture proportion, the properties of M-30R_25_ are considered as the control mix and the relative growth rate of other mixtures relative to M-30R_25_ is defined as R(%). R_C_ is the growth ratio of compressive strength, R_F_ is for flexural strength, R_Cl-_ is for chloride resistance, R_Dry_ is for dry shrinkage resistance, and R_Emi_ is for CO_2_ emission. In addition, a negative value indicates that the performance of the test mixture is inferior to that of M-30R_25_ and a positive value indicates that it is superior to that of M-30R_25_. The index R_Com_ is defined as the weighted average of the five indicators (R_C_, R_F_, R_Cl-_, R_Dry_, and R_Emi_), which is considered to comprehensively analyze the performance of eco-efficient mortar. The weight of each indicator is the value of priority in Table 6.

**Figure 9:**
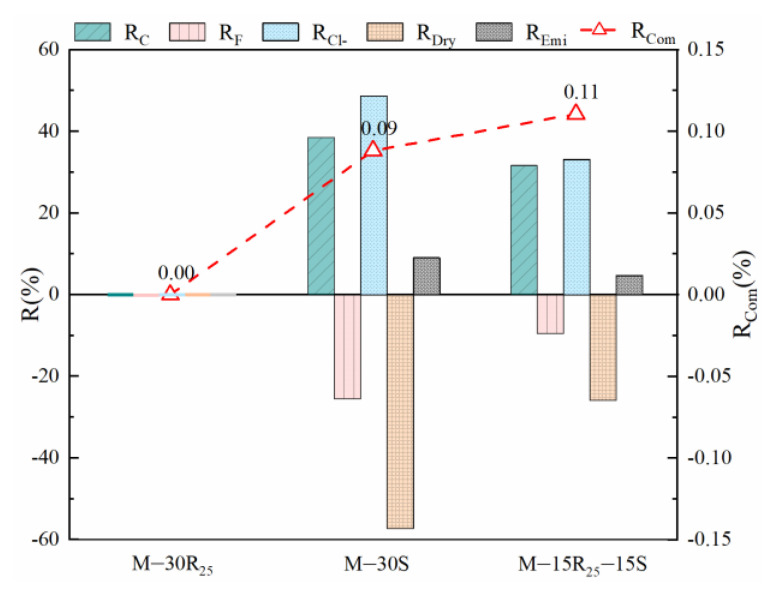
Fundamental properties of eco-efficient mortar in Series A.

The fundamental properties of eco-efficient mortar in Series A appear in Figure 9 in the order M-15R_25_-15S > M-30S > M-30R_25_. The improvements in the compressive strength and chloride resistance of M-30S are quite outstanding, but the adverse effects of SCGP on the flexural strength and dry shrinkage are also significant. Meanwhile, in terms of compressive strength and chloride resistance, M-15R_25_-15S behaves relatively well, and although the flexural strength and the shrinkage resistance are insufficient, the loss is not prominent resulting from the incorporation of an appropriate amount of RCP. Compared with other properties, the effect of SCM composition on carbon emission is not obvious, as the substitution rate of SCM is only 30%. Consequently, fundamental analysis results in this study indicate that using RCP + SCGP is better than using a single SCM (RCP or SCGP).

**Figure 10:**
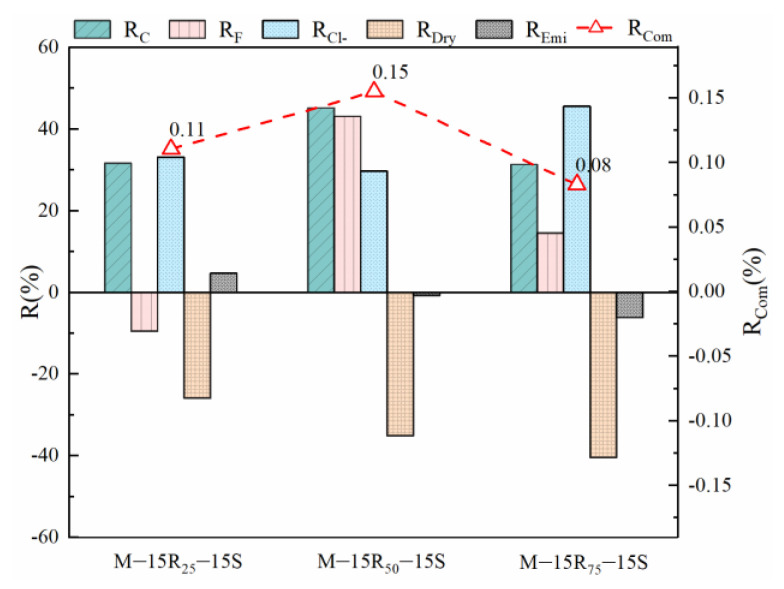
Fundamental properties of eco-efficient mortar in Series B.

Research also shows that the finely grinding process will improve the reactivity of RCP and aggravate its microaggregate effect [31]. However, the grinding process will lead to an increase in energy consumption and CO_2_ emission. The fundamental properties of eco-efficient mortar in Series B are illustrated in Figure 10, which shows that the improvement in the fundamental properties of M-15R_50_-15S makes it a better option compared to the control mix. As shown in Figure 10, the increase in grinding time has different effects on different properties. Therefore, the consideration of the fundamental performance of the mortar needs to be combined with the engineering needs and further analysis is needed.

## 4. Conclusions

This study analyzed the fundamental performance of eco-efficient mortar, including mechanical properties, long-term properties, and carbon emissions. Combined with the AHP model, optimal use of recycled powder has been analyzed, and the following conclusions are obtained:

- Attributed to the acceptable pozzolanic activity of SCGP, the mortar containing SCGP shows high compressive strength and chloride resistance. The incorporation of RCP contributes to improving the flexural strength and dry shrinkage resistance of mortar, which results from the filling ability of RCP. The eco-efficient mortar containing 15% RCP + 15% SCGP shows relatively good mechanical and long-term properties, which benefit from the synergetic effect of RCP and SCGP.
- The grinding process leads to a little improvement in the pozzolanic activity. The improvement of RCP powder fineness will be limited when the grinding time exceeds 50 min. Compared to RCP ground for under 25 min, increasing the grinding time to 50 min is beneficial to the mechanical properties of mortar but unfavorable to the long-term properties. The fusion and aggregation between RCP particles observed after 75 min of grinding lead to inferior properties of M-15R_75_-15S compared to M-15R_50_-15S.
- Among all binder materials, cement production produces the highest CO_2_ emissions. Influenced by the relatively immature production process of RCP, the CO_2_ emission from M-30S is less than that from M-30R_25_. Although inferior to SCGP, RCP ground for 75 min still emits 11.07% lower CO_2_ than cement production. Realizing the reuse of solid waste as substitutes for cement will be the appropriate method to reduce carbon emissions in the construction industry.
- Through the AHP model, the priority of various influencing factors, including mechanical properties, long-term performance, and carbon emission, on the fundamental performance is determined. As per the fundamental evaluation of this paper, M-15R_50_-15S performs the best. For different engineering applications, the evaluation priority needs to be adjusted to achieve the optimal use of solid waste.

## Figures and Tables

**Table 1:** Properties of cementitious materials (PC, SCGP, and RCP).

Material	Chemical Composition (%)								Physical Properties	
	SiO_2_	CaO	Al_2_O_3_	Fe_2_O_3_	K_2_O	SO_3_	TiO_2_	Na_2_O	Specific Surface Area (m^2^/g)	Density (g/cm^3^)
PC	17.77	63.88	7.03	5.41	0.97	1.54	0.68	0.16	10.96	3.15
SCGP	52.64	0.96	42.2	0.99	0.71	0.68	1.3	0.13	8.23	2.72
RCP	37.01	24.22	13.09	9.86	2.75	1.41	1.03	0.71	13.17	3.15

Abbreviations: PC: Portland cement; SCGP: spontaneous combustion gangue powder; RCP: recycled concrete powder.

**Table 2:** Factors and levels of the experimental design.

No.	Factor	Level 1	Level 2	Level 3
A	SCMs	RCP	SCGP	RCP + SCGP
B	RCP grinding time	25 min	50 min	75 min

Abbreviations: SCMs: supplementary cementitious materials; SCGP: spontaneous combustion gangue powder; RCP: recycled concrete powder.

**Table 3:** Mix proportions of eco-efficient mortar.

Mix No	Mix ID	W/B	Sand (kg/m^3^)	OPC (kg/m^3^)	RCP (kg/m^3^)	SCGP (kg/m^3^)
RM1	M-30R_25_	0.4	1309	493.5	211.5	0
RM2	M-30S	0.4	1294	488	0	209
RM3	M-15R_25_-15S	0.4	1301	490	105	105
RM4	M-15R_50_-15S	0.4	1301	490	105	105
RM5	M-15R_75_-15S	0.4	1301	490	105	105

Abbreviations: M: mortar; R: recycled concrete powder; S: spontaneous combustion gangue powder.

**Table 4:** CO_2_ emission components for raw materials. Reproduced with permission from [5]. Copyright John Wiley and Sons, 2020.

Item	Cement	RCP (25, 50, 75 min)	SCGP	Sand
Raw material transport (kg/t)	0.678	6.400	6.400	0.422
Solid waste landfill (kg/t)	0	1.055	1.055	0
Solid waste transport (kg/t)	0	4.523	4.523	0

**Table 5:** The fundamental scale by Saaty. Reproduced with permission from [76]. Copyright Elsevier, 1987.

Intensity of Importance on an Absolute Scale	Definition
1	Equal importance
3	Moderate importance
5	Obviously important
7	Strong importance
9	Extremely important
2, 4, 6, 8	Intermediate values between the two adjacent judgments
Reciprocal	a_ji_ = 1/a_ij_

**Table 6:** Priority of different factors.

Factors	Compressive Strength	Flexural Strength	Chloride Resistance	Dry Shrinkage	Carbon Emissions	Priority
Compressive strength	1	5	3	2	2	0.379
Flexural strength	1/5	1	1/3	1/4	1/4	0.056
Chloride resistance	1/3	3	1	1/2	1/2	0.127
Dry shrinkage	1/2	4	2	1	1	0.219
Carbon emissions	1/2	4	2	1	1	0.219

## Data Availability

Not applicable.
